# Oral health awareness and behaviors in professional orchestra musicians in Germany: findings from an online survey study

**DOI:** 10.1186/s12903-026-09381-5

**Published:** 2026-07-21

**Authors:** Felix Marschner, Annette Wiegand

**Affiliations:** Department of Preventive Dentistry, Periodontology and Cariology, University Medical Center Göttingen, Robert-Koch-Str. 40, Göttingen, 37075 Germany

**Keywords:** Musician, Orchestra, Oral health, Occupational health, Orofacial pain, Awareness

## Abstract

**Background:**

Professional musicians are exposed to occupational stressors that may affect oral health. Data on oral health awareness, preventive behavior, and instrument-related complaints among professional musicians in Germany are limited. This study aimed to investigate oral health awareness, behaviors, risk perception, and orofacial pain among orchestra musicians in Germany and to identify differences between instrument groups (woodwind, brass, string, percussion, and other instruments).

**Methods:**

An online questionnaire was sent in November 2025 to all 129 German professional orchestras. The questionnaire collected sociodemographic data, self-reported oral health, awareness of instrument-related oral health risks, professional dental care, dental care behaviors, and orofacial pain. Statistical analysis was performed by Kruskal-Wallis-tests with post-hoc Dunn’s-tests, and chi-square-tests. Multiple backward-regression models were performed for orofacial pain and awareness of instrument-related risks (*p* < 0.05).

**Results:**

494 musicians (52.8% male) were included. Most participants reported good or very good self-rated oral health (75.2%). Orofacial pain was reported by 37.1%, with significant differences between instrument groups, as string players reported pain more frequently than percussion and other instrumentalists (*p* = 0.006). Awareness of instrument-related oral health risks was high (74.2%) but varied across groups, while 79.0% felt poorly informed. 53.2% considered oral health important for professional performance. Annual dental visits and professional cleanings were reported by 44.7% and 39.1%, respectively. Preventive behaviors like toothbrushing and fluoride use were common, whereas the use of mouthguards differed by instrument, being higher among woodwind players than brass players (*p* = 0.002). Awareness of instrument-related risks was strongly associated with orofacial pain (OR = 5.41, 95%-CI:3.11–9.42) and ≥ 10 years of service (OR = 1.97, 95%-CI:1.20–3.25). Orofacial pain was associated with female gender (OR = 1.91, 95%-CI:1.28–2.86), poor self-rated oral health (OR = 2.60, 95%-CI:1.64–4.12), and awareness of instrument-related risks (OR = 6.20, 95%-CI:3.45–11.12), while musicians with ≥ 10 years of service had a lower risk (OR = 0.44, 95%-CI:0.27–0.71).

**Conclusions:**

German professional orchestra musicians report good or very good self-rated oral health and high awareness of instrument-related oral health risks, while frequently experiencing orofacial pain and feeling insufficiently informed about instrument-related oral health risks. Structured, career-long education and targeted preventive interventions are needed to reduce orofacial pain and support musicians’ long-term performance.

**Trial registration:**

ClinicalTrials.gov-NCT07008677, 28.05.2025.

**Supplementary Information:**

The online version contains supplementary material available at 10.1186/s12903-026-09381-5.

## Background

Germany has the highest density of professional orchestras worldwide [1]. According to data from the German Federal Statistical Office, more than 9,900 musicians are employed across 129 professional orchestras in Germany [2]. Professional musicians are exposed to artistic challenges, as well as various occupational stressors [3, 4], which can impact their mental and physical health [5–8], including oral health [9]. Oral health awareness and behavior represent a challenge within this occupational group [9–11]. Previous studies suggest that targeted preventive strategies, such as regular dental check-ups and the use of instrument-specific dental guards, may mitigate the negative effects of occupational stressors on oral health [12–17].

The literature suggests that playing wind instruments, compared to other instrument groups (e.g., string and percussion instruments), can lead to specific oral consequences due to the interaction between orofacial musculature and playing technique [15, 18]. Playing a wind instrument has been associated with malocclusions and changes in dentofacial morphology [19–21], temporomandibular disorders [22–24], changes in the position of anterior teeth [22, 23], and root resorption [25–27]. In this context, it can be hypothesized that wind instrument players possess higher risk awareness and preventive behavior, as orofacial complaints are more prevalent in this group.

Currently, there is a lack of data on the oral health status, preventive behaviors, and oral health awareness among professional musicians in Germany. Research differentiating between instrument groups (woodwinds, brass, strings, percussion, and other instruments such as piano, harp, or celesta) is lacking. Considering these specific occupational stressors, oral health status, oral health awareness, and behavior within this professional group remain an underexplored challenge.

This online survey aims to investigate oral health awareness and behavior among professional musicians in German orchestras and to identify potential differences between instrument groups (woodwinds, brass, strings, percussion, and other instruments such as piano, harp, or celesta). Specifically, the study aims to assess the level of oral health awareness within this professional group, to determine whether certain musician groups exhibit higher risk perception and preventive behavior, and to evaluate the implementation of preventive measures. Furthermore, the study will investigate potential variables associated with oral health awareness and orofacial pain in this population. It was hypothesized that oral health awareness, preventive behavior, and orofacial pain would differ between instrument groups, with the null hypothesis assuming no such differences.

## Methods

This online survey was performed between September and November 2025 and is reported in accordance with the Strengthening the Reporting of Observational Studies in Epidemiology (STROBE) guidelines for cross-sectional studies (Supplemental material S1) [28]. The study protocol was approved by the ethics committee of the Medical Center Göttingen (2/5/25) and was registered at ClinicalTrials.gov (NCT07008677). The study followed the Declaration of Helsinki [29].

### Study design

An online questionnaire was sent via email to the orchestral offices of all 129 professional orchestras in Germany. Additionally, the professional association and labor union for orchestra musicians, the German Orchestra Union (unisono, Berlin, Germany), distributed the online survey to all its members, inviting them to participate. After six weeks, a reminder was sent via email to the orchestral offices and through the newsletter to unisono members.

The online survey was conducted using a web-based survey platform (www.evasys.de), as described in a previous study [4]. All musicians were informed about the aim of the study and the anonymous nature of the survey. Participation was voluntary, and informed consent to participate was obtained through voluntary completion and submission of the online questionnaire.

### Participants

Musicians aged 18 or older who are employed by or freelance in a German professional orchestra are eligible to participate, while those under 18 or unwilling to give consent are excluded.

### Questionnaire

The questionnaire was used to collect data on participants’ sociodemographic characteristics: age, gender, years of service, and instrument group (woodwind instrument, brass instrument, string instrument, percussion instrument, and other instruments like piano, harp, celesta, etc.). Further questions included self-rated oral health, frequency of dental visits and professional dental cleaning, awareness of instrument-related oral health risks, importance of oral health for professional performance, and information level on oral health risks. Participants were also asked about self-reported oral health-related complaints related to playing their instrument, dental care behavior (toothbrushing frequency, fluoride product use, mouth rinse use, mouthguard/instrument-specific guard use), and interest in further preventive information.

Self-reported awareness of instrument-related risks was assessed to capture musicians’ subjective perception of potential oral health risks associated with their instrument, while information level referred to self-assessed knowledge that participants had about specific oral health risks. The questionnaire was developed with professional orchestra musicians and was pretested, and item wording was revised accordingly. The questionnaire was not formally validated, and all data were self-reported by the musicians.

### Statistic

All statistical analyses were carried out using IBM SPSS Statistics for Macintosh (Version 29.0.2.0; IBM Corp.). The level of significance was set to *p* < 0.05. As this online survey aimed to include all eligible musicians in the 129 German professional orchestras, no a priori sample size calculation was performed. The primary outcomes were orofacial pain and awareness of instrument-related risks. Secondary outcomes were self-rated oral health, frequency of dental visits and professional dental cleaning, importance of oral health for professional performance, information level on oral health risks, dental care behavior, and other self-reported oral health-related complaints (e.g., tooth displacement or tooth wear), which were also analyzed by instrument group. Interest in further preventive information was additionally recorded. Descriptive statistics were used to characterize oral health awareness, risk perception, and preventive behavior, group comparisons were used to test for differences between instrument groups, and logistic regressions were used to identify variables associated with orofacial pain and awareness of instrument-related risks. Analyses were based on available data and valid responses for each item. Descriptive data were summarized as percentages (%). Normality was evaluated using Kolmogorov–Smirnov and Shapiro-Wilk tests, both indicating non-normally distributed data. Levene’s test assessed homoscedasticity and suggested a violation of variance homogeneity. Differences in ordinal outcomes between instrument groups were evaluated using the Kruskal-Wallis test, with post-hoc Dunn’s test and Bonferroni correction for multiple comparisons. Nominal and dichotomous variables were analyzed using chi-square and Fisher’s exact tests when expected cell counts were low. Logistic regression analyses were conducted to examine predictors of orofacial pain (any pain = 1, no pain = 0) and awareness of instrument-related risks (yes = 1, no = 0). The following independent variables were included: gender (female = 1, male = 0), age (≥ 40 years = 1, < 40 years = 0), wind instrument (woodwind/brass = 1, string/percussion/other instrument = 0), years of service (> 10 years = 1, ≤ 10 years = 0), self-rated oral health (poor/very poor = 1, good/very good/average = 0), and dental visits (every 2 years/less frequently than 2 years/only in case of symptoms = 1, every 3 months/every 6 months/once per year = 0). Variables were included in a multiple backward regression model using a p-to-exit criterion of *p* = 0.10. At each step, the variable with the weakest association with the outcome was removed, and this process continued until only variables with *p* < 0.05 remained in the final model. Model performance was evaluated using Nagelkerke R² and McFadden R² for logistic regression. The number of events per variable exceeded 10 in both logistic regression models, indicating an adequate sample size and a low risk of overfitting [30].

## Results

**Fig. 1:**
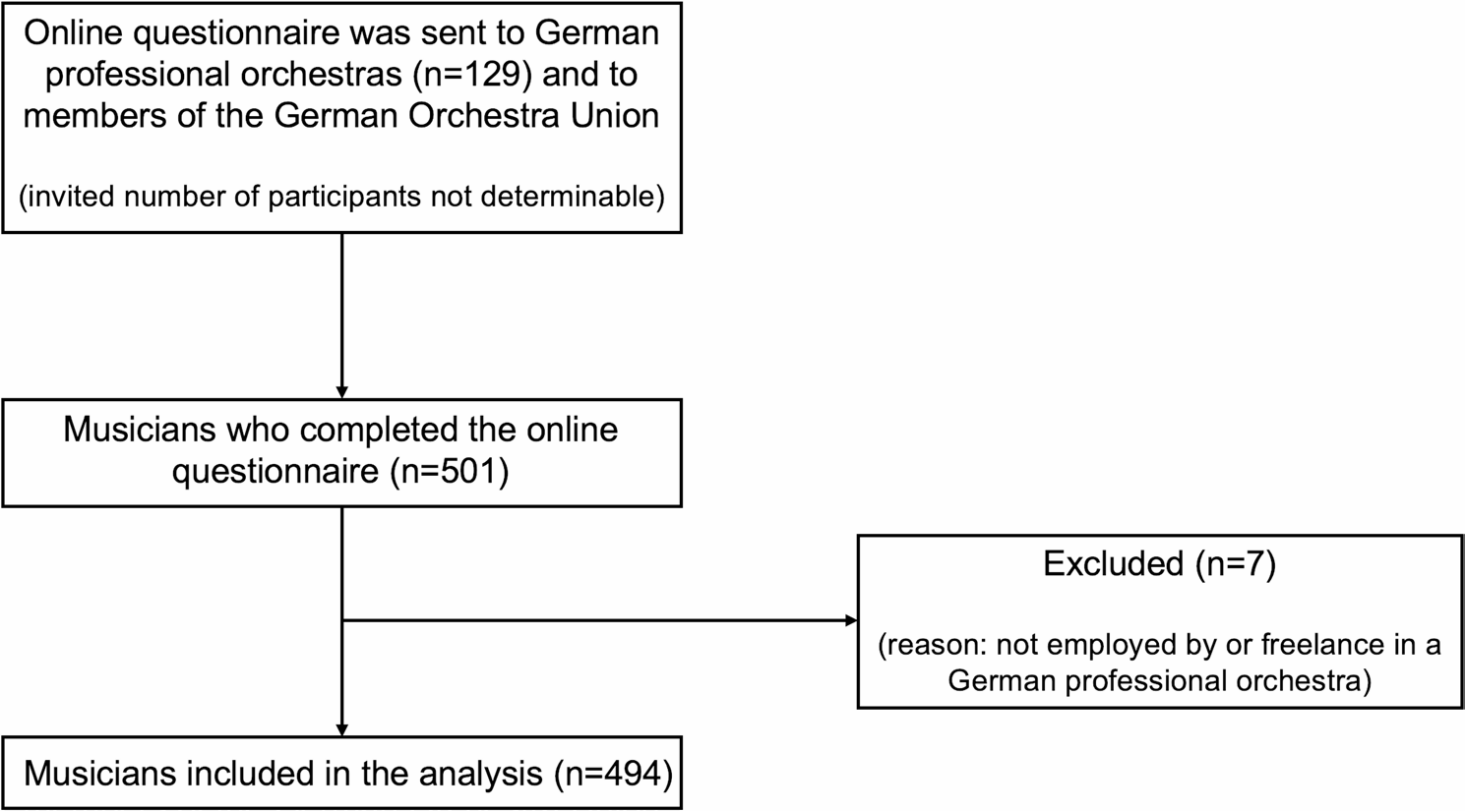
Flowchart of the recruitment process of German orchestra musicians

A total of 501 musicians participated in this online-based survey, while *n* = 494 met the inclusion criteria. The recruitment process is shown in Fig. 1. Table 1 shows sociodemographic characteristics of the study population.

**Table 1:** Sociodemographic characteristics of the study population

Sociodemographic characteristics	(*N* = 494)
Gender (%)	
Female	47.0
Male	52.8
Prefer not to say	0.2
Age groups (%)	
< 30 years	9.9
30–39 years	21.5
40–49 years	21.5
50–59 years	31.6
≥ 60 years	15.6
Years of service (%)	(*n* = 492)
< 5 years	8.1
5–10 years	14.8
11–20 years	19.7
> 20 years	57.3
Instrument group (%)	
Woodwind	35.2
Brass	26.1
String	34.8
Percussion and other^a^	3.8

^a^Other instruments like piano, harp, and celesta

In general, most of the musicians reported good or very good self-rated oral health (75.2%). Percussion and other musicians reported less frequent professional dental cleanings compared to woodwind players (p_adj_.=0.030). Overall, awareness of instrument-related oral health risks was high among all musicians (74.2%), and 53.2% indicated that oral health is important for professional performance. Musicians were generally not well-informed, with 79.0% reporting being poorly informed or uninformed about oral health risks. Characteristics for self-reported oral health, professional dental care, awareness, and level of information on oral health risks in the study population and by instrument group are presented in Table 2.

**Table 2:** Self-reported oral health, professional dental care, awareness in the study population and by instrument group

Characteristics	Total	Instrument group				
	(*N* = 494)	Woodwind (*n* = 174)	Brass (*n* = 129)	String (*n* = 172)	Percussion and other^a^ (*n* = 19)	*p*-value
Self-rated oral health (%)	(*n* = 493)			(*n* = 171)		0.370
Very good	30.6	31.0	31.0	31.6	15.8	
Good	44.6	45.4	48.8	40.4	47.4	
Average	21.1	19.0	17.1	25.1	31.6	
Poor	3.4	4.0	3.1	2.9	5.3	
Very poor	0.2	0.6	0	0	0	
Dental visits (%)						0.156
Every 3 months	3.2	4.6	3.1	2.3	0	
Every 6 months	44.1	48.3	38.8	44.8	36.8	
Once per year	44.7	40.8	47.3	45.3	57.9	
Every 2 years	2.8	2.9	3.9	1.7	5.3	
Less frequently than 2 years	1.8	1.1	1.6	2.9	0	
Only in case of symptoms	3.2	2.8	5.4	2.9	0	
Professional dental cleaning (%)						0.023*
Every 3 months	2.0	2.9	1.6	1.7	0	
Every 6 months	33.2	38.5	29.5	32.0	21.1	
Once per year	39.1	35.1	40.3	43.6	26.3	
Every 2 years	6.1	5.7	4.7	7.0	10.5	
Less frequently than 2 years	10.5	8.6	14.7	9.3	10.5	
Never	9.1	9.2	9.3	6.4	31.6	
Awareness of instrument-related oral health risks (%)	(*n* = 492)	(*n* = 173)	(*n* = 128)			0.023*
No	25.8	23.1	32.0	21.5	47.4	
Yes	74.2	76.9	68.0	78.5	52.6	
Importance of oral health for professional performance (%)	(*n* = 493)			(*n* = 171)		< 0.001*
Very important	53.3	67.2	73.6	28.1	15.8	
Important	27.4	28.2	23.3	32.7	0	
Less important	13.4	3.4	3.1	25.7	63.2	
Not important	5.9	1.1	0	13.5	21.1	
Information level on oral health risks (%)	(*n* = 491)	(*n* = 172)		(*n* = 171)		0.015*
Very well informed	3.9	3.5	6.2	2.3	5.3	
Well informed	17.1	20.9	17.8	13.5	10.5	
Poorly informed	43.4	45.3	45.0	39.8	47.4	
Not informed	35.6	30.2	31.0	44.4	36.8	

^a^Other instruments like piano, harp, and celesta

*Statistical significance (*p* < 0.05)

Orofacial pain was reported by 37.1% of the musicians, with a significant difference between string and percussion/other instrument musicians (*p* = 0.006). 13.8% of the sample reported an impact of orofacial pain on performance. Additionally, 39.9% of participants indicated tooth displacement or tooth wear, whereas percussionists and other instrumentalists reported no related complaints. Regarding performance impact, significant differences emerged only in comparisons involving the percussion and other instrument group (woodwind: p_adj_.=0.007; brass: p_adj_.<0.001; strings: p_adj_.=0.003). Self-reported oral-health-related complaints in the study population and by instrument group are reported in Table 3.

**Table 3:** Self-reported oral-health-related complaints in the study population and by instrument group

Characteristics	Total	Instrument group				
	(*N* = 494)	Woodwind (*n* = 174)	Brass (*n* = 129)	String (*n* = 172)	Percussion and other^a^ (*n* = 19)	*p*-value
Orofacial pain (%)						0.006*
No	63.0	66.1	63.6	55.8	94.7	
Occasional	29.6	26.4	29.5	35.5	5.3	
Frequent	7.5	7.5	7.0	8.7	0	
Impact of orfacial pain on performance (%)^b^						0.002*
No impact	49.7	52.4	42.7	46.0	94.1	
Mild impact	36.5	31.7	39.0	43.1	5.9	
Severe impact	11.6	12.7	17.1	8.8	0	
Very severe impact	2.2	3.2	1.2	2.2	0	
Tooth displacement or tooth wear (%)	(*n* = 489)		(*n* = 127)	(*n* = 169)		
No	60.1	59.8	58.3	57.4	100.0	0.004*
Yes	39.9	40.2	41.7	42.6	0	

^a^Other instruments like piano, harp, and celesta

^b^Only musicians reporting instrument-related orofacial pain completed this item

*Statistical significance (*p* < 0.05)

Overall, 85.8% of the musicians reported brushing their teeth several times per day, and 54.7% used fluoride products at every brushing. Mouth rinses were used daily or multiple times per day by only 16.6% of the musicians, and mouthguards were used daily by 19.9%. Regarding dental care behavior (Table 4) among musicians by instrument group, only the use of mouthguards or instrument-specific guards was statistically significant (*p* = 0.002), with woodwind musicians using guards significantly more often than brass musicians (p_adj_.<0.001).

**Table 4:** Self-reported dental care behavior among musicians in the study population and by instrument group

Characteristics	Total	Instrument group				
	(*N* = 494)	Woodwind (*n* = 174)	Brass (*n* = 129)	String (*n* = 172)	Percussion and Other^a^ (*n* = 19)	*p*-value
Use of preventive oral health measures (%)						0.295
Daily	86.2	84.5	84.5	90.1	78.9	
Several times per week	7.9	9.8	7.0	5.8	15.8	
Occasionally	5.5	5.7	7.8	3.5	5.3	
No	0.4	0	0.8	0.6	0	
Toothbrushing frequency (%)	(*n* = 493)			(*n* = 171)		0.836
Several times daily	85.8	86.8	86.0	85.4	78.9	
Daily	13.6	12.6	12.4	14.6	21.1	
Every other day	0.2	0.6	0	0	0	
Less than every other day	0.4	0	1.6	0	0	
Never	0	0	0	0	0	
Fluoride product use (%)	(*n* = 488)	(*n* = 169)		(*n* = 171)		0.446
At every brushing	54.7	56.8	45.7	57.9	68.4	
Daily	10.5	9.5	9.3	12.9	5.3	
Several times per week	6.6	6.5	7.8	6.4	0	
Occasionally	14.1	12.4	19.4	12.3	10.5	
Never	14.1	14.8	17.8	10.5	15.8	
Mouth rinse use (%)	(*n* = 488)	(*n* = 173)	(*n* = 128)	(*n* = 168)		0.904
Several times daily	4.1	4.6	3.9	4.2	0	
Daily	12.5	12.1	11.7	13.1	15.8	
Weekly	9.2	8.7	10.2	8.3	15.8	
Occasionally	26.6	29.5	25.0	26.8	10.5	
Never	47.5	45.1	49.2	47.6	57.9	
Mouthguard/instrument-specific guard use (%)	(*n* = 483)	(*n* = 173)	(*n* = 127)	(*n* = 164)		0.002*
Daily	19.9	30.6	11.8	15.2	15.8	
Several times per week	3.1	1.2	4.7	4.3	0	
As needed	6.6	6.9	4.7	7.9	5.3	
Never	70.4	61.3	78.7	72.6	78.9	

^a^Other instruments like piano, harp, and celesta

*Statistical significance (*p* < 0.05)

The multiple regression model showed that self-reported awareness of instrument-related risks was significantly associated with the presence of orofacial pain (odds ratio [OR] = 5.41, 95%-confidence interval [CI]: 3.11–9.42; *p* < 0.001), and ≥ 10 years of service (OR = 1.97, 95%-CI: 1.20–3.25; *p* = 0.008). Table 5 presents the OR for single regression models and multiple regression model.

**Table 5:** Logistic regression predicting self-reported awareness of instrument-related risks, for each removed variable, p-to-exit is reported

Variables (reference)	Single regression models				Multiple regression model		
				*p*-to-exit			
	OR	95%-CI	*p*-value		OR	95%-CI	*p*-value
Gender (male)	1.40	0.93–2.10	0.110	0.576	–	–	–
Age (< 40 years)	1.28	0.83–1.96	0.263	0.994	–	–	–
wind instrument (string/percussion/other instrument)	0.86	0.57–1.31	0.485	0.718	–	–	–
Years of service (≤ 10 years)	1.47	0.93–2.33	0.102		1.97	1.20–3.25	0.008*
Self-rated oral health (good/very good/average)	0.81	0.52–1.29	0.378	0.053	–	–	–
Dental visits (every 3 months/every 6 months/once per year)	0.50	0.25–1.00	0.049*	0.111	–	–	–
Orofacial pain (no)	4.89	2.85–8.38	< 0.001*		5.41	3.11–9.42	< 0.001*

Multiple regression model: Nagelkerke R²=0.14, McFadden R²=0.09

*Abbreviation*: *OR* Odds ratio, *CI* Confidence interval.

*Statistical significance (*p* < 0.05)

Female gender was significantly associated with orofacial pain in the multiple logistic regression model (OR = 1.91, 95%-CI: 1.28–2.86; *p* = 0.002). Musicians with ≥ 10 years of service had a lower likelihood of reporting orofacial pain (OR = 0.44, 95%-CI: 0.27–0.71; *p* < 0.001), whereas poorer self-rated oral health (OR = 2.60, 95%-CI: 1.64–4.12; *p* < 0.001) and lack of awareness of instrument-related risks (OR = 6.20, 95%-CI: 3.45–11.12; *p* < 0.001) were associated with an increased risk for orofacial pain. Further information is provided in Table 6.

**Table 6:** Logistic regression predicting instrument-related self-reported orofacial pain, for each removed variable, p-to-exit is reported

Variables (reference)	Single regression models				Multiple regression model		
				*p*-to-exit			
	OR	95%-CI	*p*-value		OR	95%-CI	*p*-value
Gender (male)	1.91	1.32–2.76	< 0.001*		1.91	1.28–2.86	0.002*
Age (< 40 years)	0.60	0.41–0.89	0.011*	0.467	–	–	–
wind instrument (string/percussion/other instrument)	0.80	0.55–1.16	0.233	0.674	–	–	–
Years of service (≤ 10 years)	0.53	0.34–0.80	0.003*		0.44	0.27–0.71	< 0.001*
Self-rated oral health (good/very good/average)	2.16	1.43–3.28	< 0.001*		2.60	1.64–4.12	< 0.001*
Dental visits (every 3 months/every 6 months/once per year)	1.20	0.62–2.34	0.592	0.726	–	–	–
Awareness of instrument-related risks (no)	4.89	2.85–8.38	< 0.001*		6.20	3.45–11.12	< 0.001*

Multiple regression model: Nagelkerke R²=0.21, McFadden R²=0.13

*Abbreviation*: *OR* Odds ratio, *CI* Confidence interval.

*Statistical significance (*p* < 0.05)

Regarding interest in further information, around half of the musicians expressed a need for guidance on dental and jaw protection (51.6%) and regular check-ups or preventive examinations (44.9%), while 31.6% sought information on measures against playing-related pain and 41.3% on specialized aids for musicians.

## Discussion

This study analyzed self-reported oral health awareness, status, and behavior among professional musicians in German orchestras and provides one of the first comprehensive data in this field. Although most musicians reported generally good self-rated oral health, they demonstrated high awareness, but limited knowledge of instrument-specific risks, and orofacial pain was a common condition.

Most musicians were aware of instrument-related oral health risks but had a low information level. This can be explained by the fact that, although complaints in daily work are frequently discussed in the orchestra pit and in personal conversations among musicians, in-depth information is often lacking. This is particularly understandable given the age structure of German orchestras, where most musicians are over 50 years old and did not receive formal training in musicians’ health during their education [31–33]. A self-reported prevalence of 37.1% of participants with orofacial pain indicates that professional music performance is associated with a considerable occupational burden. This prevalence is consistent with findings from a recent study in a similar population (woodwind and cello players in German professional orchestras) [4]. Regarding instrument groups, percussionists and players of other instruments reported fewer complaints than all other groups. Woodwind and brass musicians experienced higher prevalence, consistent with previous studies linking wind instrument playing to malocclusions, temporomandibular disorders, and tooth displacement [19, 20, 22, 34]. String musicians, especially upper‑string players, also experience substantial physical strain due to poor posture, repetitive movements, and sustained isometric muscle load [35–37].

Higher awareness of instrument-related risks was associated with orofacial pain, likely because affected musicians become more sensitized. Moreover, musicians with greater professional experience showed higher awareness of occupational risks, suggesting that such awareness develops over time and is less common among early-career musicians [38, 39]. Multiple regression analysis further identified female gender, poor self-rated oral health, ≤ 10 years of service, and awareness of instrument-related risks as significant predictors of orofacial pain. These findings suggest that, beyond instrument-specific mechanical factors, individual characteristics and awareness play a crucial role in the development of orofacial pain. The higher prevalence of orofacial pain in female musicians is in line with studies reporting greater orofacial pain and temporomandibular disorders [40, 41], not only because of hormonally mediated differences in pain perception and coping strategies but also likely due to additional biological and psychosocial mechanisms [42, 43]. These multifactorial influences may help explain the higher prevalence of orofacial pain observed among female musicians in our study. The association with poor self-rated oral health corresponds to studies linking poorer self-rated oral health to increased temporomandibular symptoms and orofacial pain, supporting its role as a predictor of orofacial pain in our study population [44, 45]. In our study, self-reported orofacial pain was also associated with a lower number of years in service. Less experienced musicians often must perform more frequently, particularly during the 18-month probation period under high psychological pressure. As a result, their cumulative exposure and workload are higher, and the risk of complaints increases with the number of performances, as reported in previous studies [7, 46].

Interestingly, higher awareness of instrument-related risks was associated with orofacial pain. A likely explanation is reverse causality. Musicians experiencing pain may become more attentive to potential risks, rather than awareness, preventing symptoms [47, 48].

Most musicians reported regular toothbrushing and use of fluoride products. 79.0% of musicians considered themselves poorly informed or uninformed. Professional dental cleaning was less frequent among percussionists and other instrumentalists, who also perceived oral health as less critical for professional performance, highlighting disparities in preventive practices across instrument groups.

These findings have practical implications for occupational health interventions among professional orchestra musicians. The results indicate that less experienced musicians tend to have a higher risk of orofacial pain and lower awareness of related risks. These findings highlight the importance of incorporating music health education as an integral part of professional training from the outset [31, 33]. Targeted educational programs should aim to reinforce knowledge of preventive measures across all career stages. Early and continuous training on oral health, instrument-specific risk prevention, and the use of protective devices could help reduce the incidence of orofacial pain and support long-term professional performance. The expressed interest in preventive guidance among participants (e.g., dental and jaw protection, regular check-ups, measures against playing-related pain) suggests high receptivity to structured interventions.

Limitations of the study included the self-reported and anonymous nature of the data, which may introduce recall or reporting bias. Moreover, the use of a study-specific, non-validated questionnaire may have introduced measurement bias. Because the questionnaire was distributed indirectly via the orchestral offices and the German Orchestra Union (unisono), the number of musicians who received the invitation cannot be determined, and a response rate cannot be calculated. Nonresponse bias cannot be excluded, as musicians with particularly high awareness of this topic may have been more willing to participate in this study. Furthermore, clinical examinations were not performed, limiting objective assessment of orofacial conditions and oral health. Besides these limitations, this study has several strengths. It is one of the first systematic surveys of oral health status and awareness among professional musicians across all 129 orchestras in Germany and was conducted in collaboration with unisono, the German Orchestra Union, the largest and most important professional association and labor union representing musicians in Germany. Nearly all professional musicians are members.

## Conclusion

This study provides one of the first comprehensive assessments of oral health awareness, behavior, and orofacial pain among professional orchestra musicians in Germany. Together with the interest in preventive guidance expressed by the participants, the findings emphasize the need for structured, career-spanning educational programs. Whether such education and preventive measures reduce orofacial pain and support long-term professional performance should be examined in future studies.

## Supplementary Information

Supplementary Material 1.

## Data Availability

All data generated or analyzed during this study are included in this published article. Further enquiries can be directed to the corresponding author.

## References

[1] German Orchestra Union (unisono). Unisono statistics: established posts and classification of professional orchestras. German Orchestra Union (unisono). 2024. https://uni-sono.org/klassikland-deutschland/statistik-planstellen-einstufung-berufsorchester/. Accessed: 18 Dec 2025.

[2] Liersch A, Asef D. Music sector report 2016. Federal Statistical Office, Education and Culture 2017. https://www.destatis.de/DE/Themen/Gesellschaft-Umwelt/Bildung-Forschung-Kultur/Kultur/Publikationen/Downloads-Kultur/spartenbericht-musik-5216203169004.pdf?__blob=publicationFile. Accessed: 18 Dec 2025.

[3] Vastamäki M, Heliövaara M, Vastamäki H, Ristolainen L. Orchestra musicians’ work environment and health versus general workforce. J Occup Environ Med. 2023;65:344–48. 10.1097/jom.0000000000002763. 36730011 10.1097/JOM.0000000000002763

[4] Marschner F, Sokolowski A, Sokolowski A, Biermann J, Wiegand A. Orofacial pain and oral health-related quality of life in woodwind and cello musicians in german orchestras: An online based questionnaire study. J Occup Med Toxicol. 2025;20:19. 10.1186/s12995-025-00467-4. 40481603 10.1186/s12995-025-00467-4 PMC12143053

[5] Voltmer E, Zander M, Fischer JE, Kudielka BM, Richter B, Spahn C. Physical and mental health of different types of orchestra musicians compared to other professions. Med Probl Perform Art. 2012;27:9–14. 22543317

[6] Rodríguez-Gude C, Taboada-Iglesias Y, Pino-Juste M. Musculoskeletal pain in musicians: Prevalence and risk factors - a systematic review. Int J Occup Saf Ergon. 2023;29:883–901. 10.1080/10803548.2022.2086742. 35678565 10.1080/10803548.2022.2086742

[7] Gómez-Rodríguez R, Díaz-Pulido B, Gutiérrez-Ortega C, Sánchez-Sánchez B, Torres-Lacomba M. Prevalence, disability and associated factors of playing-related musculoskeletal pain among musicians: A population-based cross-sectional descriptive study. Int J Environ Res Public Health. 2020;17. 10.3390/ijerph17113991. 10.3390/ijerph17113991 PMC7312771 32512798

[8] Okoshi K, Minami T, Kikuchi M, Tomizawa Y. Musical instrument-associated health issues and their management. Tohoku J Exp Med. 2017;243:49–56. 10.1620/tjem.243.49. 28931767 10.1620/tjem.243.49

[9] Bergström J, Eliasson S. Dental care habits, oral hygiene, and gingival health in swedish professional musicians. Acta Odontol Scand. 1985;43:191–7. 10.3109/00016358509046498. 3864337 10.3109/00016358509046498

[10] Herman E. Dental considerations in the playing of musical instruments. J Am Dent Assoc. 1974;89:611–9. 10.14219/jada.archive.1974.0433. 4528504 10.14219/jada.archive.1974.0433

[11] Bergström J, Eliasson S. Dental health in professional musicians. A radiographic study in dental conscious subjects. Swed Dent J. 1985;9:225–31. 3866339

[12] Nii M, Yoda N, Putra RH, Aida J, Sasaki K. Evaluation of the optimal hardness and thickness of music splints for wind instrument players. J Prosthet Dent. 2023;129:754–62. 10.1016/j.prosdent.2021.06.044. 34364689 10.1016/j.prosdent.2021.06.044

[13] Wilson JS. A dental appliance for a clarinettist experiencing temporomandibular joint pain. Med Probl Perform Art. 1989;4:118–21.

[14] Katada C, Imai M, Nozaki K, Kawamoto M, Maeda Y, Shima Y. Investigation of the ton modification after inserting music splint into oral cavity by the digital filtering technique. Jpn J Med Inf. 2005;25:231–38.

[15] Czech NP, Alt KW. Wind instruments and oral health: Challenges faced by professional wind musicians. Dent J (Basel). 2024;12. 10.3390/dj12100306. 10.3390/dj12100306 PMC11505960 39452434

[16] van der Weijden F, Berkhout FRU, Lobbezoo F. Improvement of embouchure after correction of irregular front teeth: The case of a professional french horn player. Br Dent J. 2019;226:261–64. 10.1038/s41415-019-0013-4. 30796395 10.1038/s41415-019-0013-4

[17] Clemente MP, Moreira A, Carvalho N, et al. Orofacial trauma on the anterior zone of a trumpet’s player maxilla: Concept of the oral rehabilitation-a case report. Int J Environ Res Public Health. 2020;17. 10.3390/ijerph17249423. 10.3390/ijerph17249423 PMC7765605 33339137

[18] Bouros E, Protogerou V, Castana O, Vasilopoulos G. Respiratory function in wind instrument players. Mater Sociomed. 2018;30:204–08. 10.5455/msm.2018.30.204-208. 30515060 10.5455/msm.2018.30.204-208 PMC6195392

[19] Glória JC, Balestra AA, Iasbik NS, Douglas-de-Oliveira DW, Flecha OD, Gonçalves PF. Prevalence of orofacial changes in wind instrumentalists: A cross-sectional pilot study in brazil. Med Probl Perform Art. 2018;33:1–5. 10.21091/mppa.2018.1002. 29600302 10.21091/mppa.2018.1002

[20] van der Weijden FN, Kuitert RB, Lobbezoo F, Valkenburg C, van der Weijden GA, Slot DE. Does playing a wind instrument influence tooth position and facial morphology? Systematic review and meta-analysis. J Orofac Orthop. 2020;81:267–85. 10.1007/s00056-020-00223-9. 32556368 10.1007/s00056-020-00223-9 PMC7316676

[21] Macovei G, Minea R, Dumitraș IT, Precup CA, Baroiu L, Nechifor A, Armencia AO, Lese AC. Changes in dento-facial morphology induced by wind instruments, in professional musicians and physical exercises that can prevent or improve them-a systematic review. Life (Basel). 2023;13. 10.3390/life13071528. 10.3390/life13071528 PMC10381963 37511903

[22] Głowacka A, Matthews-Kozanecka M, Kawala M, Kawala B. The impact of the long-term playing of musical instruments on the stomatognathic system - review. Adv Clin Exp Med. 2014;23:143–6. 10.17219/acem/37038. 24596017 10.17219/acem/37038

[23] Gotouda A, Yamaguchi T, Okada K, Matsuki T, Gotouda S, Inoue N. Influence of playing wind instruments on activity of masticatory muscles. J Oral Rehabil. 2007;34:645–51. 10.1111/j.1365-2842.2007.01765.x. 17716263 10.1111/j.1365-2842.2007.01765.x

[24] Yeo DK, Pham TP, Baker J, Porters SA. Specific orofacial problems experienced by musicians. Aust Dent J. 2002;47:2–11. 10.1111/j.1834-7819.2002.tb00296.x. 12035952 10.1111/j.1834-7819.2002.tb00296.x

[25] Shafi I, Welbury R. Idiopathic radiographic apical root resorption in wind instrument players. Dent Update. 2015;42:972–6. 10.12968/denu.2015.42.10.972. 26856005 10.12968/denu.2015.42.10.972

[26] Seres L, Vetro E, Perenyi J, Kocsis A. Severe root resorption of the upper central incisors as a consequence of playing the flute. Dent Traumatol. 2017;33:406–09. 10.1111/edt.12352. 28602035 10.1111/edt.12352

[27] Gunst V, Huybrechts B, De Almeida Neves A, Bergmans L, Van Meerbeek B, Lambrechts P. Playing wind instruments as a potential aetiologic cofactor in external cervical resorption: Two case reports. Int Endod J. 2011;44:268–82. 10.1111/j.1365-2591.2010.01822.x. 21166826 10.1111/j.1365-2591.2010.01822.x

[28] von Elm E, Altman DG, Egger M, Pocock SJ, Gøtzsche PC, Vandenbroucke JP. Strengthening the reporting of observational studies in epidemiology (strobe) statement: Guidelines for reporting observational studies. BMJ. 2007;335:806–8. 10.1136/bmj.39335.541782.AD. 17947786 10.1136/bmj.39335.541782.AD PMC2034723

[29] World medical association. Declaration of helsinki: Ethical principles for medical research involving human subjects. JAMA. 2013;310:2191–4. 10.1001/jama.2013.281053. 24141714 10.1001/jama.2013.281053

[30] Peduzzi P, Concato J, Kemper E, Holford TR, Feinstein AR. A simulation study of the number of events per variable in logistic regression analysis. J Clin Epidemiol. 1996;49:1373–9. 10.1016/s0895-4356(96)00236-3. 8970487 10.1016/s0895-4356(96)00236-3

[31] Matei R, Broad S, Goldbart J, Ginsborg J. Health education for musicians. Front Psychol. 2018;9:1137. 10.3389/fpsyg.2018.01137. 30061850 10.3389/fpsyg.2018.01137 PMC6055059

[32] Altenmüller E, Kopiez R, Grewe O, Schneider S, Eschrich S, Nagel F, Jabusch HC. The institute for music physiology and musicians’ medicine. Cogn Process. 2007;8:201–6. 10.1007/s10339-007-0174-y. 17624562 10.1007/s10339-007-0174-y

[33] Matei R, Ginsborg J. Health education for musicians in the uk: A qualitative evaluation. Health Promot Int. 2022;37. 10.1093/heapro/daab146. 10.1093/heapro/daab146 34562098

[34] Masiulytė V, Žarovienė A, Švalkauskienė V. Orthodontic problems among string and wind instrument players. Stomatol. 2021;23:41–7. 34528907

[35] Ahlberg J, Wiegers JW, van Selms MKA, Peltomaa M, Manfredini D, Lobbezoo F, Savolainen A, Tuomilehto H. Oro-facial pain experience among symphony orchestra musicians in finland is associated with reported stress, sleep bruxism and disrupted sleep-independent of the instrument group. J Oral Rehabil. 2019;46:807–12. 10.1111/joor.12818. 31081155 10.1111/joor.12818

[36] Z’Graggen S, Ettlin DA, Alessandri E, Z’Graggen WJ, Schimmel M. Prevalence of painful temporomandibular disorder symptoms among professional and student musicians: An online survey. J Oral Rehabil. 2025;52:9–16. 10.1111/joor.13868. 39344421 10.1111/joor.13868 PMC11680494

[37] van Selms MKA, Wiegers JW, van der Meer HA, Ahlberg J, Lobbezoo F, Visscher CM. Temporomandibular disorders, pain in the neck and shoulder area, and headache among musicians. J Oral Rehabil. 2020;47:132–42. 10.1111/joor.12886. 31520546 10.1111/joor.12886 PMC7004094

[38] Bena A, Giraudo M, Leombruni R, Costa G. Job tenure and work injuries: A multivariate analysis of the relation with previous experience and differences by age. BMC Public Health. 2013;13:869. 10.1186/1471-2458-13-869. 24053157 10.1186/1471-2458-13-869 PMC3849089

[39] Breslin FC, Dollack J, Mahood Q, Maas ET, Laberge M, Smith PM. Are new workers at elevated risk for work injury? A systematic review. Occup Environ Med. 2019;76:694–701. 10.1136/oemed-2018-105639. 31147382 10.1136/oemed-2018-105639

[40] Bueno CH, Pereira DD, Pattussi MP, Grossi PK, Grossi ML. Gender differences in temporomandibular disorders in adult populational studies: A systematic review and meta-analysis. J Oral Rehabil. 2018;45:720–29. 10.1111/joor.12661. 29851110 10.1111/joor.12661

[41] Häggman-Henrikson B, Liv P, Ilgunas A, et al. Increasing gender differences in the prevalence and chronification of orofacial pain in the population. Pain. 2020;161:1768–75. 10.1097/j.pain.0000000000001872. 32701837 10.1097/j.pain.0000000000001872 PMC7365674

[42] Khan A, Liu S, Tao F. Mechanisms underlying sex differences in temporomandibular disorders and their comorbidity with migraine. Brain Sci. 2024;14. 10.3390/brainsci14070707. 10.3390/brainsci14070707 PMC11274652 39061447

[43] Yap AU, Lei J, Liu CG, Huang ZW, Fu KY. Sex-related differences in temporomandibular disorder symptom severity: Correlates with jaw function and oral health-related quality of life among patients. J Oral Rehabil. 2025. 10.1111/joor.70103. 41239755 10.1111/joor.70103

[44] Kojima A, Ekuni D, Mizutani S, et al. Relationships between self-rated oral health, subjective symptoms, oral health behavior and clinical conditions in japanese university students: A cross-sectional survey at okayama university. BMC Oral Health. 2013;13:62. 10.1186/1472-6831-13-62. 24195632 10.1186/1472-6831-13-62 PMC4228361

[45] Yamane-Takeuchi M, Ekuni D, Mizutani S, et al. Associations among oral health-related quality of life, subjective symptoms, clinical status, and self-rated oral health in japanese university students: A cross-sectional study. BMC Oral Health. 2016;16:127. 10.1186/s12903-016-0322-9. 27903265 10.1186/s12903-016-0322-9 PMC5129632

[46] Paarup HM, Baelum J, Holm JW, Manniche C, Wedderkopp N. Prevalence and consequences of musculoskeletal symptoms in symphony orchestra musicians vary by gender: A cross-sectional study. BMC Musculoskelet Disord. 2011;12:223. 10.1186/1471-2474-12-223. 21978278 10.1186/1471-2474-12-223 PMC3221643

[47] González-Roldán AM, Bustan S, Kamping S, Flor H, Anton F. Pain and related suffering reduce attention toward others. Pain Pract. 2023;23:873–85. 10.1111/papr.13260. 37296080 10.1111/papr.13260

[48] Menéndez-Torre Á, Martin-Pintado-Zugasti A, Paris-Alemany A, Bocos-Corredor E, Molina-Álvarez M, Arribas-Romano A, Fernández-Carnero J. Pain sensitization and pain-related psychological factors in patients with temporomandibular disorders: An observational cross-sectional study. Clin Oral Investig. 2024;28:594. 10.1007/s00784-024-05954-2. 39400763 10.1007/s00784-024-05954-2

